# Sitagliptin and oral cancer risk in type 2 diabetes patients

**DOI:** 10.18632/oncotarget.18239

**Published:** 2017-05-27

**Authors:** Chin-Hsiao Tseng

**Affiliations:** ^1^ Department of Internal Medicine, National Taiwan University College of Medicine, Taipei, Taiwan; ^2^ Division of Endocrinology and Metabolism, Department of Internal Medicine, National Taiwan University Hospital, Taipei, Taiwan; ^3^ Division of Environmental Health and Occupational Medicine of the National Health Research Institutes, Zhunan, Taiwan

**Keywords:** diabetes mellitus, oral cancer, sitagliptin, Taiwan

## Abstract

The reimbursement database of the Taiwan’s National Health Insurance was used to evaluate oral cancer risk after sitagliptin use. Patients newly diagnosed of type 2 diabetes during 1999–2008 were recruited. A 1:1 propensity score matched-pair sample of 39195 ever users and 39195 never users were followed up until December 31, 2011. Cox regression incorporated with the inverse probability of treatment weighting using propensity score was used to estimate hazard ratios. Results showed that the overall hazard ratio was not statistically significant (0.956, 95% confidence interval: 0.652–1.401). However, in tertile analyses, the hazard ratio for the first (< 7.47 months), second (7.47–15.63 months) and third (> 15.63 months) tertile of cumulative duration was 1.563 (0.963–2.537), 1.236 (0.738–2.071) and 0.345 (0.164–0.725), respectively; and was 1.575 (0.963–2.575), 1.224 (0.738–2.033) and 0.347 (0.165–0.731), respectively, for the first (< 19,600 mg), second (19,600–42,200 mg) and third (> 42,200 mg) tertile of cumulative dose. Sensitivity analyses after excluding patients who developed any other cancer during follow-up did not change the results substantially. Additionally, the risk of oral diseases that may predispose to oral cancer (i.e., “gingival and periodontal diseases" and/or "oral mucosal lesions") paralleled the risk pattern of oral cancer, suggesting a possible explanation for the risk change of oral cancer related to sitagliptin. In conclusion, sitagliptin may reduce oral cancer risk when the cumulative duration is > 15.63 months or the cumulative dose is > 42,200 mg.

## INTRODUCTION

Incretin-based therapies by using the oral form of dipeptidyl peptidase-4 (DPP-4) inhibitors have become a mainstay in the treatment of type 2 diabetes mellitus. Sitagliptin, probably the most commonly used DPP-4 inhibitor, was the first in the class approved for clinical use in 2006 [1]. There are some concerns on cancer risk related to the use of incretin-based therapies, especially for pancreatic cancer and thyroid cancer [1–3]. On the other hand, some animal and *in vitro* studies suggested that sitagliptin inhibits the growth of colorectal cancer [1, 4]. A Japanese case report showed that sitagliptin treatment for 3 weeks dramatically regressed hepatocellular carcinoma in a patient with hepatitis C infection [5]. In a recent observational study, sitagliptin reduced the risk of prostate cancer [6].

Whether sitagliptin may increase or decrease the risk of oral cancer has not been investigated. The present study evaluated such risk after sitagliptin use in type 2 diabetes patients by using the reimbursement records in Taiwan’s National Health Insurance (NHI) database. Other incretin-based therapies (including saxagliptin, vildagliptin, linagliptin and alogliptin for DPP-4 inhibitors; and exenatide and liraglutide for glucagon-like peptide 1 receptor agonists) currently available in Taiwan were not evaluated because they were not approved until after mid-2010 [1] and therefore were less commonly used during the study period.

## RESULTS

**Figure 1:**
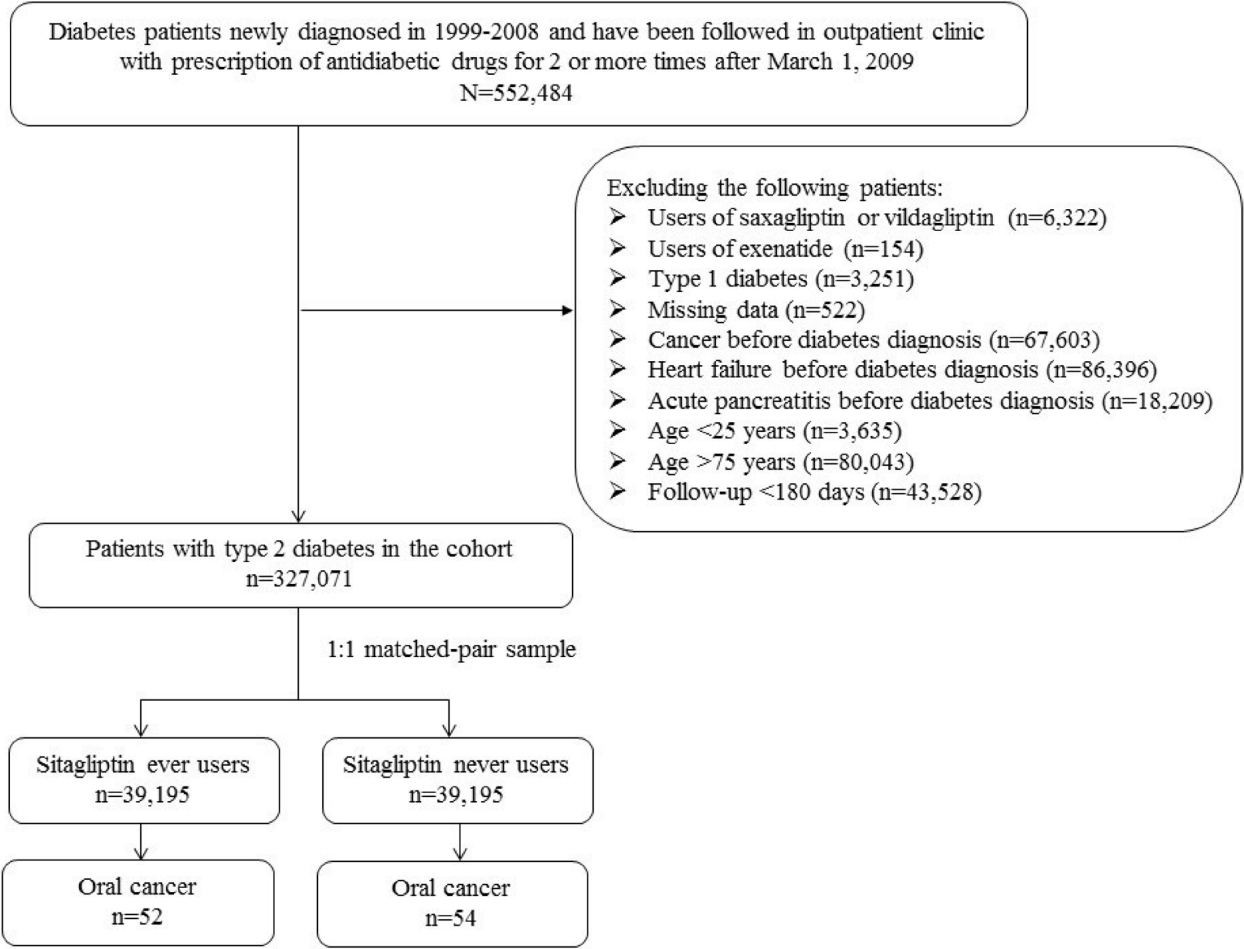
Flowchart showing the procedure in selecting a 1:1 matched-pair sample into the study from the reimbursement database of the National Health Insurance

There were 39195 never users and 39195 ever users in the matched cohort (Figure 1). Although some variables (i.e., age, hypertension, statin, angiotensin converting enzyme inhibitor/angiotensin receptor blocker, calcium channel blocker, sulfonylurea, insulin, acarbose, rosiglitazone, aspirin and dipyridamole) differed significantly, none had a value of standardized difference > 10% (Table 1). Therefore, the results were unlikely influenced by residual confounding from the differences in these characteristics.

**Table 1:** Characteristics of never users and ever users of sitagliptin

Variable	Never users (*n =* 39195)		Ever users (*n =* 39195)		*P*-value	SD
	*n*	%	*n*	%		
Age (years)	55.81 ± 10.16		56.19 ± 9.97		< 0.0001	3.83
Diabetes duration (years)	6.60 ± 2.74		6.63 ± 2.79		0.1764	0.53
Sex (men)	21331	54.42	21282	54.30	0.7253	−0.16
Hypertension	30121	76.85	30434	77.65	0.0077	1.90
Chronic obstructive pulmonary disease	16590	42.33	16639	42.45	0.7232	0.20
Stroke	9056	23.10	9086	23.18	0.7994	0.14
Nephropathy	8821	22.51	8940	22.81	0.3100	0.81
Ischemic heart disease	14965	38.18	14881	37.97	0.5367	−0.37
Peripheral arterial disease	8005	20.42	8162	20.82	0.1658	1.09
Eye disease	12766	32.57	12785	32.62	0.8849	0.12
Obesity	2955	7.54	2826	7.21	0.0779	−1.47
Dyslipidemia	32800	83.68	32978	84.14	0.0836	1.39
Acute pancreatitis	198	0.51	201	0.51	0.8803	0.10
Tobacco abuse	1405	3.58	1491	3.80	0.1034	1.21
Alcohol−related diagnoses	2134	5.44	2071	5.28	0.3179	−0.68
Gingival and periodontal diseases	35017	89.34	34974	89.23	0.6195	−0.38
Oral mucosal lesions	866	2.21	841	2.15	0.5407	−0.53
Statin	27403	69.91	27767	70.84	0.0044	2.11
Fibrate	16169	41.25	16420	41.89	0.0689	1.47
Angiotensin converting enzyme inhibitor/angiotensin receptor blocker	27046	69.00	27534	70.25	0.0002	2.68
Calcium channel blocker	20072	51.21	20550	52.43	0.0006	2.47
Sulfonylurea	21967	56.05	23149	59.06	< 0.0001	5.85
Metformin	29817	76.07	30018	76.59	0.0912	0.82
Insulin	2350	6.00	2186	5.58	0.0121	−2.23
Acarbose	4444	11.34	4257	10.86	0.0335	−1.60
Pioglitazone	2588	6.60	2577	6.57	0.8742	0.22
Rosiglitazone	1770	4.52	1453	3.71	< 0.0001	−4.07
Aspirin	21560	55.01	21938	55.97	0.0066	1.89
Ticlopidine	1253	3.20	1323	3.38	0.1608	0.99
Clopidogrel	3098	7.90	3016	7.69	0.2748	−0.81
Dipyridamole	12029	30.69	12387	31.60	0.0058	2.10

Age and diabetes duration are expressed as mean ± standard deviation

SD: standardized difference

Table 2 shows the incidence rates of oral cancer and hazard ratios by sitagliptin exposure. The respective incident number of oral cancer for never users and ever users was 54 and 52, with respective incidence of 75.68 and 72.38 per 100,000 person-years. The overall hazard ratio of 0.956 (95% confidence interval: 0.652–1.401) suggested a null association. However, when evaluating the distribution of the incident cases of oral cancer by the tertiles of cumulative duration and cumulative dose of sitagliptin therapy, there was a trend of decreasing incidence with longer duration or higher cumulative dose. A significantly reduced risk of approximately 65% was observed for the third tertiles. The results in sensitivity analyses after excluding patients who developed any other cancer during follow-up were very similar and did not change the conclusion of the study.

**Table 2:** Incidence rates of oral cancer and hazard ratios by sitagliptin exposure

Sitagliptin use	Cases followed	Incident cases of oral cancer	Person-years	Incidence rate (per 100,000 person-years)	Hazard ratio	95% Confidence interval	*P*-value
Never users	39195	54	71353.08	75.68	1.000		
Ever users	39195	52	71839.78	72.38	0.956	(0.652−1.401)	0.8173
**Cumulative duration (months)**							
Never users	39195	54	71353.08	75.68	1.000		
< 7.47	13324	24	21264.36	112.86	1.563	(0.963−2.537)	0.0708
7.47−15.63	12533	20	21879.55	91.41	1.236	(0.738−2.071)	0.4204
> 15.63	13338	8	28695.87	27.88	0.345	(0.164−0.725)	0.0050
**Cumulative dose (mg)**							
Never users	39195	54	71353.08	75.68	1.000		
< 19,600	12716	23	20366.50	112.93	1.575	(0.963−2.575)	0.0703
19,600−42,200	13135	21	22845.36	91.92	1.224	(0.738−2.033)	0.4335
> 42,200	13344	8	28627.92	27.94	0.347	(0.165−0.731)	0.0053
**Sensitivity analyses after excluding patients who developed any other cancer during follow−up**							
Never users	38256	52	69499.80	74.82	1.000		
Ever users	38180	49	69916.09	70.08	0.938	(0.634−1.388)	0.7486
**Cumulative duration (months)**							
Non−users	38256	52	69499.80	74.82	1.000		
< 7.47	12927	22	20617.51	106.71	1.496	(0.905−2.471)	0.1161
7.47−15.63	12223	20	21296.44	93.91	1.284	(0.764−2.157)	0.3445
> 15.63	13030	7	28002.15	25.00	0.313	(0.142−0.689)	0.0040
**Cumulative dose (mg)**							
Non−users	38256	52	69499.80	74.82	1.000		
< 19,600	12326	21	19725.73	106.46	1.503	(0.902−2.505)	0.1175
19,600−42,200	12824	21	22266.16	94.31	1.269	(0.762−2.113)	0.3595
> 42,200	13030	7	27924.19	25.07	0.316	(0.143–0.695)	0.0042

Table 3 shows the incidence rates of oral diseases including “gingival and periodontal diseases” and/or “oral mucosal lesions” and the hazard ratios by sitagliptin exposure. The findings paralleled those observed for oral cancer and suggested an overall neutral effect. Although the risk was significantly higher for the first and second tertiles of cumulative duration and cumulative dose, the risk reduced to a significant level in patients categorized in the third tertiles.

**Table 3:** Incidence rates of oral diseases and hazard ratios by sitagliptin exposure

Sitagliptin use	Cases followed	Incident cases of oral diseases	Person-years	Incidence rate (per 100,000 person-years)		Hazard ratio		95% Confidence interval		*P*-value	
Never users	4764	610	8169.96	7466.38		1.000					
Ever users	4764	616	8275.77	7443.41		0.999		(0.892−1.118)		0.9797	
**Cumulative duration (months)**							
Non-users	4764	610	8169.96	7466.38		1.000					
< 6.87	1573	278	2385.93	11651.65		1.634		(1.416−1.885)		< 0.0001	
6.87–14.93	1532	214	2437.67	8778.89		1.214		(1.038−1.419)		0.0154	
> 14.93	1659	124	3452.18	3591.94		0.446		(0.368−0.542)		< 0.0001	
**Cumulative dose (mg)**							
Non-users	4764	610	8169.96	7466.38		1.000					
< 17,600	1569	269	2376.27	11320.26		1.591		(1.377−1.838)		< 0.0001	
17,600–40,500	1574	222	2544.72	8723.96		1.198		(1.027−1.399)		0.0217	
> 40,500	1621	125	3354.79	3726.02		0.464		(0.383−0.563)		< 0.0001	

Oral diseases include “gingival and periodontal diseases” and/or “oral mucosal lesions”

## DISCUSSION

This is the first observational study conducted in humans to evaluate the risk of oral cancer after sitagliptin use in type 2 diabetes patients. Although the risk was neither increased nor decreased in the overall analyses, a significantly lower risk was seen in the third tertiles of cumulative duration and cumulative dose in either the primary analyses or the sensitivity analyses (Table 2). The risk of oral diseases that may predispose to oral cancer (i.e., “gingival and periodontal diseases” and/or “oral mucosal lesions”) seemed to echo the findings observed for oral cancer (Table 3).

The mechanisms of a reduced risk of oral cancer after sitagliptin use remains to be explored. A recent study suggested that the activity of DPP-4 in the saliva of patients with periodontitis was significantly elevated and positively correlated with all clinical parameters of periodontitis including the prevalence of infection with *Porphyromonas gingivalis* [7]. Chronic inflammation is a key component of tumor progression in the oral cavity [8]. Diabetes patients suffer from a significantly higher risk of periodontitis [9, 10] and oral cancer [11]. Therefore, a potential mechanism linking a reduced risk of oral cancer after sitagliptin use is through its inhibition of the activity of DPP-4 in oral mucosa. The findings in Table 3 showing a significantly reduced risk of oral diseases after prolonged use of sitagliptin provided a support for this explanation. Anagliptin, a DPP-4 inhibitor, facilitated the restoration of mucosal damage in a model of experimental murine colitis [12]. An *in vitro* study also showed promising anti-cancer activity of sitagliptin and vildagliptin on colon cancer cell lines (HT-29), with sitagliptin more potent than vildagliptin [13]. Sitagliptin also corrected the dysbiosis of gut microbiota in a rat model [14]. Although DPP-4 activity in the oral mucosa of patients with oral cancer remains unknown, it might also be high as observed in patients with periodontitis [7]. It is worthwhile to investigate such enzyme activity in these patients and to see whether DPP-4 inhibitors may prevent the development of or have a therapeutic role on oral cancer.

Smoking, alcohol drinking and betel nut chewing are recognized risk factors of oral cancer [15, 16]. However, we were not able to evaluate their potential confounding effects due to the lack of information in the NHI database. A confounder should both be correlated with the exposure (sitagliptin use) and the outcome (oral cancer), and should not be an intermediate between exposure and outcome [17]. Because of the lack of significant difference in the distribution of chronic obstructive pulmonary disease (a surrogate for smoking), tobacco abuse and alcohol-related diagnoses between ever and never users of sitagliptin (Table 1), and because betel nut chewers are always smokers in Taiwan [18–20], we have no reason to believe that these factors can play important role as confounders.

Human papillomavirus may also play an important role in oral cancer [21], especially in women [22]. However, this infection was not considered in the analysis because only 8 patients were identified with such a diagnosis. The small case number did not allow any analysis with sufficient power. Therefore, potential confounding effect of this viral infection requires further investigation.

The higher risk of oral cancer (not significant, Table 2) and oral diseases (significant, Table 3) for the first and second tertiles deserves some discussion. Sitagliptin is a relatively novel drug and not recommended as a first-line treatment in Taiwan. Patients who started with sitagliptin might have higher glucose levels not satisfactorily managed by other preexisting antidiabetic drugs. Therefore, confounding by indication related to a poorer glycemic control in new users of sitagliptin in the first and second tertiles is possible. Biochemical data relevant to glycemic control were not available in the NHI database and such a possibility awaits further clarification.

This study has several strengths. The database covers almost the whole population and keeps all claims records from outpatient visits and hospital admission. Therefore, the findings in the present study can be readily generalized to the whole population. With the use of medical records, self-reporting bias can also be limited. The present study also suffers from less bias from different detection rates of oral cancer or other comorbidities among different social classes because of the following reasons. First, the Bureau of NHI considers cancer as a severe morbidity and patients with a cancer diagnosis are waived of most medical co-payments. Second, the drug cost-sharing is low or can be waived for patients with certain conditions including low-income household, veterans and prescription refills for chronic disease.

Study limitations may include a lack of actual measurement data of smoking and alcohol drinking, and the lack of information of other confounders such as betel nut chewing, lifestyle, diet, family history, and genetic parameters. The impact of glycemic control and the role of the pathology, grading and staging of oral cancer could not be evaluated because these data are not available in the database. Finally, whether the findings of the present study can be generalized to other DPP-4 inhibitors or to glucagon-like peptide 1 receptor agonists require further investigation.

In summary, this study suggests that sitagliptin use may reduce the risk of oral cancer after a cumulative duration of > 15.63 months or a cumulative dose of > 42,200 mg. In parallel, the risk of oral diseases that may predispose to oral cancer also decreases after prolonged use of sitagliptin. Because of the observational nature of the study, additional confirmation is necessary.

## MATERIALS AND METHODS

The NHI healthcare system and its reimbursement database in Taiwan have been described in detail in previously published papers [23, 24]. In brief, the NHI is compulsory and covers > 99% of the population and has contracts with > 98% of all hospitals in Taiwan. The database can be used for academic research after review and approval. This study was granted with an approval number 99274.

Disease diagnoses are coded by the *International Classification of Diseases, Ninth Revision, Clinical Modification* (ICD-9-CM) during the study period; and diabetes was coded 250.XX and oral cancer 140, 141, 143, 144, 145, 146, 148, and 149.

Figure 1 shows the procedure in selecting a cohort of 1:1 propensity score (PS) matched-pair sample of sitagliptin ever and never users. Patients were newly diagnosed of type 2 diabetes mellitus at the age of 25–74 years from 1999 to 2008. They should have received prescriptions of antidiabetic drugs for 2 or more times at the outpatient clinic until after March 1, 2009 (the date sitagliptin was approved for reimbursement by the Bureau of NHI). Patients with a diagnosis of diabetes mellitus during 1996–1998 were excluded to assure their diabetes being first diagnosed after 1999. To avoid the potential confounding from other incretin-based therapies that were approved for clinical use during follow-up, users of saxagliptin or vildagliptin (*n =* 6322) and exenatide (*n =* 154) were excluded. A total of 522 patients were excluded due to missing data and 3251 patients with type 1 diabetes mellitus (based on the issuance of the so-called “Severe Morbidity Card” after a certified diagnosis) were excluded because incretin-based therapies were not approved for their treatment. Because incretin-based therapies have been reported to increase the risk of congestive heart failure, acute pancreatitis and cancers involving the pancreas and thyroid [1–3, 25, 26], patients who had been diagnosed of any cancer (*n =* 67603), congestive heart failure (*n =*86396) or acute pancreatitis (*n =* 18209) before entry were also excluded. Patients aged < 25 (*n =* 3635) or > 75 (*n =* 80043) years and those followed up for a duration < 180 days (*n =* 43528) were also excluded. In consideration of an imbalance in characteristics between sitagliptin ever and never users in this original sample, a 1:1 PS matched-pair sample was created using the Greedy 8 --> 1 digit match algorithm as recommended by Parsons [27]. The PS was derived from all characteristics and the date of entry by logistic regression. This matching method has been used and described in detail in our previous studies [6, 28–31].

Cumulative duration (months) and cumulative dose (mg) of sitagliptin use were calculated and their tertiles were used for analyses. Comorbidities and covariates used in the study included [32–37]: age, diabetes duration, sex, hypertension (ICD-9-CM code: 401–405), chronic obstructive pulmonary disease (a surrogate for smoking; 490–496), stroke (430–438), nephropathy (580–589), ischemic heart disease (410–414), peripheral arterial disease (250.7, 785.4, 443.81 and 440–448), eye disease (250.5, 362.0, 369, 366.41 and 365.44), obesity (278), dyslipidemia (272.0–272.4), acute pancreatitis (577.0), tobacco abuse (305.1, 649.0 and 989.84), alcohol-related diagnoses (291, 303, 535.3, 571.0–571.3, 980.0), gingival and periodontal diseases (523), and oral mucosal lesions (528.6, 528.7, 528.8). Antidiabetic drugs other than sitagliptin and medications commonly used in diabetes patients included insulin, sulfonylurea, metformin, meglitinide, acarbose, pioglitazone, rosiglitazone, statin, fibrate, angiotensin converting enzyme inhibitor/angiotensin receptor blocker, calcium channel blocker, aspirin, ticlopidine, clopidogrel and dipyridamole. These characteristics between never and ever users of sitagliptin were compared by Student’s *t* test for age and diabetes duration and by Chi-square test for other variables.

The incidence density of oral cancer was calculated for different subgroups of sitagliptin exposure, i.e., for never users, ever users and tertiles of cumulative duration and cumulative dose. The numerator of the incidence density was the case number of new-onset oral cancer during follow-up. The denominator was calculated from the person-years of follow-up, which ended at the time of a new diagnosis of oral cancer, on the date of the last reimbursement record, or on December 31, 2011.

Hazard ratios comparing different subgroups of sitagliptin exposure to never users were estimated by Cox regression incorporated with the inverse probability of treatment weighting using the PS [38]. Because sitagliptin may increase or decrease the risk of some other cancers [1–6], sensitivity analyses were conducted after excluding patients who developed other cancers during follow-up. To examine whether sitagliptin might also increase or decrease the risk of oral diseases that may predispose to oral cancer, the incidence of “gingival and periodontal diseases” and/or “oral mucosal lesions” and hazard ratios were also calculated. These analyses were conducted in a PS-matched cohort of ever and never users of sitagliptin who did not have these oral diseases at entry.

SAS statistical software, version 9.3 (SAS Institute, Cary, NC), was used for statistical analyses. A *P*-value < 0.05 was considered as statistically significant.
